# Musculoskeletal pain during and after SARS-CoV-2 infection and healthcare utilization: a cross-sectional study

**DOI:** 10.1186/s12891-023-06794-z

**Published:** 2023-08-29

**Authors:** Abelardo de Oliveira Soares Junior, Max dos Santos Afonso, Yohana Pereira Vieira, Juliana Quadros Santos Rocha, Samuel Dumith, Rosália Garcia Neves, Carine Nascimento da Silva, Suele Manjourany Silva Duro, Mirelle de Oliveira Saes

**Affiliations:** 1 Federal University of Rio Grande, Rio Grande, Rio Grande do Sul Brazil; 2 Anhanguera Faculty, Rio Grande, Rio Grande do Sul Brazil; 3 State Secretary of Health, Pelotas, Rio Grande do Sul Brazil; 4 Federal University of Pelotas, Pelotas, Rio Grande do Sul Brazil

**Keywords:** Coronavirus infections, Covid-19, Musculoskeletal pain, Health services

## Abstract

**Background:**

The COVID-19 pandemic necessitated the reorganization of health services to cater to the needs of individuals affected by the virus.

**Objective:**

This study aimed to examine the association between musculoskeletal pain during and after SARS-CoV-2 infection and the utilization of health services among adults in southern Brazil.

**Methods:**

A cross-sectional study was conducted with individuals aged 18 years and older, who were diagnosed with COVID-19 between December 2020 and March 2021. Questionnaires were electronically collected using tablets through the REDCap platform via phone calls. The health service utilization outcomes assessed included Primary Health Care, general practitioners, private emergency care, and specialized services. The exposure variable was the presence of musculoskeletal pain during and after SARS-CoV-2 infection in different regions, such as cervical, upper limbs, thoracic, lumbar, and lower limbs. Poisson regression analysis was employed to assess the relationship between health service utilization during and after SARS-CoV-2 infection and musculoskeletal pain during and after the infection with SARS-CoV-2 among adults in southern Brazil. Data were analyzed using the Stata 16.1 statistical package.

**Results:**

A total of 2,919 individuals were interviewed. Overall, individuals with musculoskeletal pain were found to utilize health services approximately 15 percentage points higher when compared to those without musculoskeletal pain. In adjusted analysis, individuals who reported musculoskeletal pain during and after SARS-CoV-2 infection were up to twice as likely to use health services. Among them, the emergency care unit was the most frequently used service, particularly in those with pain in the lower limbs (RP=2.19, 95% CI 1.66-2.87) and thoracic region (RP=2.04, 95% CI 1.47-2.84). Notably, the highest magnitudes of association were observed with emergency care units, specialist doctors, and specialized services, especially neurologists, who were two to three times more likely to be sought, followed by pulmonologists.

**Conclusion:**

Health service utilization was significantly associated with musculoskeletal pain during and after SARS-CoV-2 infection. All regions, except for the cervical region, showed a correlation with the use of Primary Health Care. The thoracic region featured an association with pulmonologists and emergency room utilization. Additionally, health services like emergency care units, specialist doctors, and specialized services, including cardiologists and neurologists, were commonly utilized across all regions in southern Brazil.

**Supplementary Information:**

The online version contains supplementary material available at 10.1186/s12891-023-06794-z.

## Introduction

The COVID-19 pandemic was a major challenge for health services worldwide, burdening them with a new, initially unknown demand [1, 2]. Due to the abrupt changes, it can be observed that services still have insufficient infrastructure in the outpatient care of people with sequelae of COVID-19 [3, 4].

Long COVID, the persistence of symptoms after the acute phase of the disease, has been widely cited in the literature, with prevalence ranging from 11% to more than 70% [5–7].

Musculoskeletal pain (MSK) is among the most frequent symptoms of long COVID, such as lumbar pain (70.7%), myalgia (61.0%), and arthralgia (44.0%) [8]. This can be explained by the virus' ability to affect the different structures that make up the musculoskeletal system, such as bones, muscles, joints, blood vessels, and peripheral nerves, which can lead to painful symptoms [8].

Nevertheless, it is still not known as there are not enough studies in the literature on the presence of MSK pain after SARS-CoV-2 infection in the use of health services, specifically. Thus, the aim of this study was to verify the relationship between the presence of MSK pain during and after infection with SARS-CoV-2 and the use of health services after infection in adults in southern Brazil.

## Methodology

### Study design

This research presents the baseline data of the Sulcovid-19 survey, an investigation tracking health indicators in adults following SARS-CoV-2 infection. The study protocol received approval from the Health Research Ethics Committee of the Federal University of Rio Grande (FURG) (CAAE: 39081120.0.0000.5324). All participants provided informed consent before taking part in the study [9].

### Setting

Rio Grande is a port city, covering 2,817 km^2^, with a population of 191,900 inhabitants [10]. For the development of the research, initially, we contacted the Epidemiological Health Surveillance of the municipality of Rio Grande to identify adults with COVID-19 during the investigated period. A list of individuals with positive RT-PCR results and their respective information (name, address, telephone number, and presence of symptoms) was prepared. The interviewees participated voluntarily in the interviews. Data collection occurred from June to October 2021. Questionnaires were collected electronically using tablets through the REDCap program, and smartphones were used for phone calls. To ensure the safety of both the researcher and the interviewee, the calls were recorded using a free mobile application (Callmarter) and stored in an email account. The questionnaire application lasted approximately 20 minutes.

### Participants

The sample included individuals aged 18 years or older, residents of the municipality of Rio Grande, diagnosed with SARS-Cov-2 through the RT-PCR test between December 2020 and March 2021. In addition, who underwent treatment in the city of Rio Grande/RS and who presented musculoskeletal symptoms such as pain/discomfort in the cervical/neck, upper limbs, lower limbs, thoracic region, lumbar region, in at least one of the regions of the spine (cervical, thoracic and lumbar) and at least one of the body regions investigated (cervical, upper limbs, thoracic, lumbar and lower limbs). Individuals with functional limitations and/or advanced neurological diseases that made it impossible to fill out the questionnaire and that did not have a caregiver to answer for them were excluded, as well as those who were in long-stay institutions or deprived of freedom (prisons). Individuals not located after five attempts of telephone contact, one via *WhatsApp*, and three home visits were considered losses and refusals.

### Variables

The outcomes use of health services were asked individually. Primary Health Care (PHC) (Unidade Básica de Saúde in Portuguese), general practitioner (medico generalista privado in Portuguese), Emergency room (Pronto socorro in portuguese) Emergency care unit (Unidade de pronto atendimento in Portuguese) were investigated through the following question “After your SARS-CoV-2 infection, how many times did you need to be seen in (health service)?” with a continuous response option (number of times the service was used), being dichotomized into no (0 visits) and yes (one or more visits).

Specialized services such as pulmonologists, neurologists, cardiologists, psychiatrists, physiotherapists, psychologists, and speech therapists, were investigated through the question “After your SARS-CoV-2 infection, you needed to seek specialized care with (you can tick as many options as you want)”: with dichotomous answer option No/Yes. As for the aggregated variables, the outcome “specialist doctors “was constructed from the combination of “pulmonologist, neurologist, cardiologist, and psychiatrist” variables. As for the outcome “specialized services” from the combination of the variables “pulmonologist, neurologist, cardiologist, psychiatrist, physiotherapist, psychologist, and speech therapist”, both outcomes were dichotomized (no/yes), considering yes, the use of at least one of the investigated services.

The exposure presence of musculoskeletal pain was assessed using questions based the Nordic questionnaire on musculoskeletal symptoms [11]. The presence of musculoskeletal pain/discomfort was evaluated during and/or after infection by COVID-19 in the following regions: cervical, upper limbs (shoulders, elbows, wrists, hands), thoracic, lumbar, and lower limbs (hips, thighs, knees, ankles, feet), based on the following questions: “Do you feel pain/discomfort? in any of the regions below?”; “Did this pain/discomfort start during or after your SARS-CoV-2 infection? Based on this, the outcomes presence of musculoskeletal pain/discomfort during/after infection in the cervical region, upper, thoracic, lumbar and lower limbs were configured, as well as the outcomes: presence of pain/discomfort in at least one of the regions of the spine (cervical, thoracic and lumbar) and at least one of the body regions investigated (cervical, upper limbs, thoracic, lumbar and lower limbs). All variables were dichotomized (no/yes).

The following covariates were used to adjustment for possible confounding: Sex (Male or Female), Age range (18-59 years old/60 years old or older), Race (white/yellow and black/brown/indigenous), Education (Never studied, 1º grade, 2º grade and 3º grade), Marital status (Married, lives with partner/Single and separated and widowed) and Income (0-1000, 1001-2000, 2001-4000 and 4000 or more in BRL). Other variables for sample description were: physical activity (Inactive: <150 minutes/week and Active: >150 minutes/week”) [12], Economic class (A/B, C and D/E), Smoking (no and yes/ex-smoker), Body mass index (Low weight/eutrophic and Overweight/obesity), Health self-perception (Very bad / bad, Moderate, Good and Very good), Hypertension (No and Yes), Anxiety (No and Yes), Depression (No and Yes), Heart problems (i.e Heart failure, cardiomegaly; No and Yes), Diabetes (No and Yes), Musculoskeletal, (i.e Arthritis, arthrosis, rheumatism or osteoporosis; No and Yes), Breathing problems (i.e Asthma, Bronchitis, Emphysema or chronic obstructive pulmonary disease; No and Yes), Multimorbidity (0, 1 or 2 and 3 or more), Hospitalization during SARS-CoV-2 infection (i.e infirmary or intensive care unit; No and Yes).

### Statistical methods

Descriptive data are presented as proportions and 95% confidence intervals (CI95%). We used Poisson regression to evaluate the relationship between healthcare services use after COVID-19 and musculoskeletal pain after infection with SARS-Cov-2 in adults in southern Brazil. Adjusted analyses were performed using Poisson regression with robust adjustment of variance. The CI95% with no overlapping between categories were considered associated. Data were analyzed using the statistical package Stata 16.1.

## Results

A total of 3,822 participants who tested positive for COVID-19 were eligible for the study; after losses (*n=*631) and refusals (*n=*272), 2,919 (76.4% of those eligible) were interviewed. The sample consisted predominantly of women (59.6%), individuals between 18 and 59 years old (83.3%), white/yellow (77.9%) and married/ lives with partner (60.6%). Regarding schooling and economic class, 44.2% had completed high school and 54.8% belonged to the highest economic class.

About 60.0% were physically inactive, 24.4% smoked, 73.3% were overweight/obese and 58.1% had a good self-perception of health. For the 43.1% who had at least one preexisting comorbidity, the most prevalent were anxiety (26.3%), and depression (19.4%). Among those investigated, only 3.7% needed to be hospitalized, causing most of the sample to be patients with a mild form of infection (Table 1).

**Table 1:** Description of the sample of individuals after SARS-CoV-2 infection, Rio Grande do Sul, Brazil, 2021 (*n=*2.919)

**Variable**	**n**	**%**
Sex		
Male	1.208	41.4
Female	1.711	59.6
Age range (years)		
18-59	2420	83.3
60 or more	482	16.7
Race		
White/yellow	2.254	77.9
Black/brown/indigenous	640	22.1
Education		
Never studied	15	0.5
1º grade	713	24.9
2º grade	1264	44.2
3º grade	871	30.4
Marital status		
Married/lives with partner	1757	60.6
Single/separated/widowed	1144	39.4
Economic class		
A/B	809	30.1
C	1474	54.7
D/E	409	15.2
Income		
0-1000	668	26.1
1001-2000	995	38.9
2001-4000	604	23.6
4000 or more	288	11.3
Physical activity^e^		
Inactive	1697	58.5
Active	1205	41.5
Smoking		
No	2197	75.6
yes/ex	708	24.4
Body mass index		
Low weight/eutrophic	757	26.7
Overweight/obesity	2076	73.3
Health self-perception		
Very bad / bad	107	3.8
Moderate	633	21.7
Good	1692	58.1
Very good	480	16.5
Hypertension		
No	2160	74.7
Yes	733	25.3
Anxiety		
No	2136	73.7
Yes	761	26.3
Depression		
No	2340	80.6
Yes	563	19.4
Heart problems^a^		
No	2630	91.0
Yes	260	9.0
Diabetes		
No	2605	89.9
Yes	292	10.1
Musculoskeletal^**b**^		
No	2489	86.7
Yes	383	13.3
Breathing problems^**c**^		
No	2409	83.1
Yes	488	16.9
Multimorbidity		
0	1058	37.4
1 or 2	1219	43.1
3 or more	549	19.4
Hospitalization during SARS-CoV-2 infection^**d**^		
No	2307	96.3
Yes	88	3.7

^a^Heart failure, cardiomegaly

^b^Arthritis, arthrosis, rheumatism or osteoporosis

^c^Asthma, Bronchitis, Emphysema or chronic obstructive pulmonary disease

^d^infirmary or intensive care unit

^e^Inactive: <150 minutes/week; Active: >150 minutes/week

**Fig. 1:**
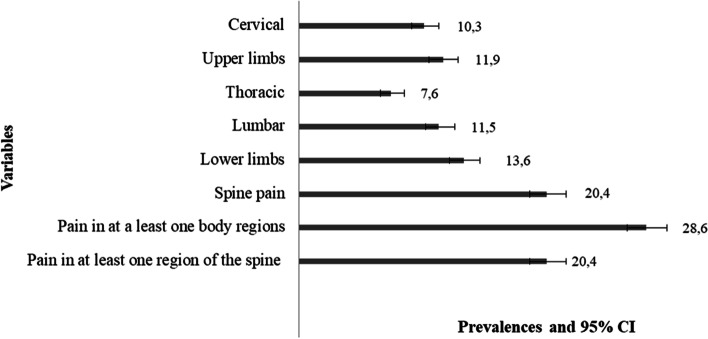
Prevalence of the presence of musculoskeletal pain during/after SARS-CoV-2 infection in individuals from Rio Grande do Sul, Brazil, 2021 (*n=* 2,919)

The prevalence of musculoskeletal pain during and after infection was 13.6% (CI95% 12.4-14.9) in the lower limbs, 11.9% (CI95% 10.7-13.1) in the upper limbs, 11.5% (CI95% 10.4-12.8) in the lumbar, 10.3% (CI95% 9.3-11.5) in the cervical and 7.6% (CI95% 6, 7-8,7) in the thoracic. In addition, the prevalence of pain in at least one of the body regions evaluated was 28.6% (CI95% 27.0-30.3) followed by pain in at least one region of the spine at 20.4% (CI95% 19.0 -22.0) (Fig. 1).

The prevalence of use of health services according to the presence of musculoskeletal pain in at least one of the main exposures in long COVID individuals was 36.5% (CI95% 33.3-39.9), followed by specialized services at 30.3% (CI95% 27.2-33.5) and regarding the prevalence of specialist doctors, cardiologists 18.5% (CI95% 16.0-21.3) and pulmonologists 8.5% (CI95% 6.7-10.6) had a higher prevalence. However, the services with less demand for care were emergency rooms at 4.3% (CI95% 3.1-5.9) (Table 2).

**Table 2:** Prevalence of use of health services according to the absence and presence of musculoskeletal pain during/after SARS-CoV-2 infection in individuals from Rio Grande do Sul, Brazil, 2021 (*n=* 2,919)

Variable	Without musculoskeletal pain	Cervical	Upper limb	Thoracic	Lumbar	Lower limbs	Spine*	Body regions**
P% (95% CI)								
Primary Health Care	27.7 (26.2-29.4)	35.7 (30.4-41.4)	37.7 (32.6-43.0)	42.4 (35.8-48.9)	40.2 (35.0-45.60	41.9 (37.1-46.9)	36.6 (32.8-40.6)	36.5 (33.3-39.9)
General Practitioner	26.3 (24.7-28.0)	29.8 (24.9-35.3)	26.2 (21.8-31.1)	26.4 (20.9-32.6)	28.3 (23.7-33.4)	32.1 (27.7-40.0)	28.1 (24.6-31.9)	27.4 (24.4-30.5)
Emergency Room	2.6 (2.1-3.3)	3.4 (1.8-6.2)	2.6 (1.4-5.0)	7.2 (4.5-11.5)	3.6 (2.0-6.2)	5.3 (3.5-8.1)	4.3 (2.9-6.2)	4.3 (3.1-5.9)
Emergency care unit	9.7 (8.7-10.9)	15.5 (11.8-20.1)	18.8 (15.0-23.3)	20.4 (15.5-26.2)	18.4 (14.6-23.0)	19.6 (15.9-23.8)	15.9 (13.2-19.1)	15.7 (13.4-18.4)
Specialist doctors	20.3 (18.9-21.8)	27.4 (22.6-32.8)	29.4 (24.8-34.5)	29.9 (24.1-36,3)	25.9 (21.4-30.9)	30.3 (25.9-35.0)	25.6 (22.3-29.3)	26.2 (23.3-29.4)
Specialized services	24.7 (23.2-26.3)	33.1 (28.0-38.7)	33.1 (28.3-38.4)	35.0 (28.9-41.6)	30.7 (25.9-35.9)	33.7 (29.1-38.5)	30.2 (26.6-34.1)	30.3 (27.2-33.5)
Pulmonologist	7.3 (6.4-8.3)	10.1 (7.1-14.2)	10.3 (7.5-14.0)	11.3 (7.7-16.2)	8.4 (5.9-12.0)	9.7 (7.1-13.0)	8.7 (6.7-11.3)	8.5 (6.7-10.6)
Cardiologist	13.7 (12.5-15.0)	17.2 (13.3-21.9)	18.5 (14.7-23.0)	22.2 (17.1-28.2)	17.7 (14.0-22.2)	21.6 (17.8-26.0)	17.7 (14.9-21.1)	18.5 (16.0-21.3)
Neurologist	3.2 (2.7-4.0)	6.1 (3.8-9.4)	6.7 (4.5-10.0)	7.2 (4.5-11.5)	6.0 (4.0-9.1)	7.6 (5.4-10.7)	5.5 (3.9-7.6)	5.3 (3.9-7.0)
Psychiatrist	3.9 (3.3-4.7)	6.4 (4.1-9.8)	7.0 (4.7-10.3)	7.2 (4.5-11.5)	6.9 (4.6-10.2)	6.4 (4.3-9.2)	6.6 (4.9-9.0)	6.1 (4.7-8.0)
Physiotherapist	3.4 (2.8-4.2)	4.4 (2.5-7.4)	5.0 (3.1-7.9)	5.4 (3.1-9.3)	3.0 (1.6-5.5)	6.4 (4.3-9.2)	3.6 (2.3-5.4)	4.2 (3.0-5.8)
Psychologist	6.3 (5.5-7.3)	9.8 (6.9-13.7)	8.5 (6.0-12.0)	11.4 (7.8-16.3)	9.6 (6.9-13.3)	8.9 (6.4-12.2)	8.9 (6.8-11.5)	8.1 (6.4-10.2)

Specialist doctors: pulmonologist, neurologist, cardiologist, and psychiatrist

Specialized services: pulmonologist, neurologist, cardiologist, psychiatrist, physiotherapist, psychologist, and speech therapist

*Spine: cervical, thoracic and lumbar

**Body regions: cervical, upper limb, thoracic, lumbar and lower limbs

In general, in our study, use of health services was about 15 percentual points (p.p) higher in individuals with musculoskeletal pain when compared with people without musculoskeletal pain. There was a higher prevalence of use of PHC, mostly for individuals with thoracic pain 42.4% (CI95% 35.8-48.9), lower limbs 41.9 % (CI95% 37.1-46.9), and in the lumbar 40.2% (CI95% 35.0-45.60). In the second place, the most service use was specialist services for thoracic (35.0% CI95% 28.9-41.6), lower limbs (33.7% CI95% 29.1-38.5), cervical (33.1% CI95% 28.0-38.7) and upper limb pain (33.1% CI95% 28.3-38.4). On the other hand, the lowest prevalence of service use was identified for emergency room which ranged from 2.6% (CI95% 1.4-5.0) in the upper limbs and 4.3% (CI95% 2.9-6.2) for spine (Table 2).

In the adjusted analysis, it was observed that individuals who reported pain in all outcomes during and after the SARS-CoV-2 infection were up to twice as likely to use health services and among them, the emergency care unit was more used, especially in those with pain in the lower limbs (PR=2.19; CI95% 1.66-2.87) and thoracic pain (PR=2.04; CI95% 1.47-2.84). Given this, the highest magnitudes of association were related to emergency care units, physiotherapist and specialized services, especially neurologists, who showed between two and three times greater probability of looking for this professional, followed by a pulmonologist, especially in those individuals with thoracic pain (PR=1.78; CI95% 1.13-2.80), in the cervical (PR=1.69; CI95% 1.14-2.51) and the upper limbs (PR=1.53; 95% CI 1.04-2.26) when compared to individuals without musculoskeletal pain during and after SARS-CoV-2 infection (Table 3).

**Table 3:** Poisson regression analysis adjusted between use of health services and musculoskeletal pain during/after SARS-CoV-2 infection in individuals from Rio Grande do Sul, Brazil, 2021 (*n=* 2,919)

**Variable**	**Cervical**	**Upper limb**	**Thoracic**	**Lumbar**	**Lower limbs**	**Spine**^**a**^	**Body regions**^**b**^
Prevalence Ratio (95%CI)							
Primary Health Care	1.21 (0.98-1.50)	**1.26 (1.02-1.53)**	**1.37 (1.09-1.73)**	**1.38 (1.13-1.67)**	**1.45 (1.21-1.75)**	**1.29 (1.09-1.52)**	**1.35 (1.16-1.58)**
General Practitioner	1.18 (0.93-1.49)	0.97 (0.76-1.24)	1.02 (0.78-1.37)	1.12 (0.89-1.40)	**1.27 (1.03-1.55)**	1.08 (0.90-1.31)	1.05 (0.89-1.24)
Emergency Room	1.14 (0.58-2.25)	0.82 (0.41-1.67)	**2.89 (1.64-5.11)**	1.13 (0.59-2.16)	**1.98 (1.17-3.35)**	1.60 (0.96-2.63)	**1.69 (1.05-2.73)**
Emergency care unit	**1.57 (1.13-2.16)**	**1.95 (1.46-2.60)**	**2.04 (1.47-2.84)**	**1.88 (1.40-2.53)**	**2.19 (1.66-2.87)**	**1.79 (1.38-2.32)**	**1.93 (1.50-2.48)**
Specialist doctors	**1.49 (1.17-1.90)**	**1.54 (1.23-1.94)**	**1.62 (1.24-2.13)**	**1.35 (1.07-1.72)**	**1.59 (1.29-2.00)**	**1.39 (1.14-1.69)**	**1.47 (1.22-1.76)**
Specialized services	**1.43 (1.15-1.79)**	**1.37 (1.11-1.70)**	**1.49 (1.16-1.92)**	**1.28 (1.03-1.60)**	**1.39 (1.14-1.70)**	**1.31 (1.09-1.57)**	**1.33 (1.12-1.56)**
Pulmonologist	**1.69 (1.14-2.51)**	**1.53 (1.04-2.26)**	**1.78 (1.13-2.80)**	1.18 (0.77-1.81)	1.40 (0.96-2.04)	1.29 (0.92-1.81)	1.23 (0.90-1.69)
Cardiologist	**1.46 (1.07-1.98)**	**1.46 (1.09-1.93)**	**1.88 (1.36-2.60)**	**1.45 (1.08-1.94)**	**1.73 (1.35-2.26)**	**1.52 (1.20-1.94)**	**1.66 (1.32-2.06)**
Neurologist	**2.26 (1.33-3.85)**	**2.40 (1.45-3.96)**	**2.57 (1.45-4.57)**	**2.07 (1.22-3.48)**	**3.01 (1.90-4.79)**	**2.06 (1.30-3.26)**	**2.20 (1.41-3.42)**
Psychiatrist	1.47 (0.88-2.46)	**1.69 (1.06-2.71)**	1.71 (0.98-2.97)	**1.62 (1.01-2.61)**	1.54 (0.97-2.43)	**1.66 (1.10-2.49)**	**1.60 (1.08-2.37)**
Physiotherapist	1.41 (0.76-2.60)	1.50 (0.85-2.65)	1.71 (0.87-3.36)	0.86 (0.42-1.73)	1.05 (1.25-3.38)	1.09 (0.65-1.84)	1.30 (0.82-2.05)
Psychologist	**1.49 (1.00-2.24)**	1.23 (0.81-1.85)	**1.70 (1.10-2.63)**	1.42 (0.95-2.10)	1.34 (0.92-1.97)	1.36 (0.97-1.91)	1.25 (0.91-1.72)

Adjusted by: sex, age, race, education, marital status and income

Specialist doctors: pulmonologist, neurologist, cardiologist, and psychiatrist

Specialized services: pulmonologist, neurologist, cardiologist, psychiatrist, physiotherapist, psychologist, and speech therapist

^a^Spine: cervical, thoracic and lumbar

^b^Body regions: cervical, upper limb, thoracic, lumbar and lower limbs

## Discussion

The present study verified the relationship between presence of musculoskeletal pain during and after infection with SARS-CoV-2 and the use of health services after infection in adults in southern Brazil. It was observed that individuals who reported pain in at least one of the main exposures eligible for this study needed to use some type of health services. Among these health services that presented the highest prevalence after the period of infection were the emergency care units, followed by PHC and specialized services. In addition, it is noted that the individuals evaluated in this study sought care from specialist doctors, with the highest prevalence being cardiologists and pulmonologists.

Given the results obtained in this study, more than half of the interviewees were from economic class C and had a low level of education, which favors the process of inequality in the use of health services, given that the latest data from the PNS confirmed the strong dependence on the population to public health services since 71.5% of people declared that they did not have access to private medical health plans [13].

In 2019, according to the PNS, the number of people who sought health services within one year, when compared to those who indicated having good or very good health, was 10.9 p.p. lower than the others in 2019, a difference smaller than those observed in 2013 and 2008 [13, 14].

According to the literature, around 10% of individuals will have musculoskeletal pain after infection with COVID-19, whether hospitalized or not [15]. Prevalence data on musculoskeletal pain after SARS-CoV-2 infection are scarce in the literature regarding patients who had the mild form of infection. A meta-analysis showed that 10% of the investigated individuals had back pain (CI 95% 1-23%; I 2 80.20%) after SARS-CoV-2 infection, but the study does not report whether only hospitalized or hospitalized individuals were evaluated [16]. Another study with individuals who were hospitalized shows similar data to our research: 19.4% of patients reported pain in the lower limbs, which was more prevalent. However, they present a lower prevalence of musculoskeletal pain in other regions: 7.9% in the upper limbs, 6.8% in the lumbar, 9.1% in the neck, and 14.1% had thoracic pain [15].

There are some hypotheses for the pain to present as a symptom after COVID-19 and it seems that it is not a single reason [17]. It is possible that damage to certain organs may be associated with infection, muscle weakness, contractures, and injuries to the somatosensory system may be some of the contributing factors to the existence of pain [18]. In addition, alterations in metabolism and hormonal balance, cytokine storm, and neuronal damage seem to contribute to the persistence of these symptoms [17]. Not much is known about why pain is present in certain locations, but lower limb pain was the most common form of musculoskeletal pain after SARS-CoV-2 infection, corroborating the findings of this study [8, 19].

Individuals with musculoskeletal pain in all regions except the cervical region were more likely to use PHC, which is in line with the literature that compared to 12 months prior to SARS-CoV-2 infection, PHC consultation rates for (non-hospitalized) patients with COVID-19 were 1.89 (CI95%1.63; 2.20) times higher after COVID-19 for muscle pain [20]. Rates of PHC use were significantly higher after COVID-19 for thoracic pain and muscle pain, relative to a negative control (patients never tested for covid-19) and influenza cohorts [20]. The PHC is able to solve 85% of the health problems of users in the community, with low technological density [21]. Musculoskeletal problems are primarily needed by physical therapy professionals [22]. Thus, the presence of the physical therapist professional in primary care, for resolution of the basic type of longitudinal22, the recovery time, and future costs with prevention [23].

Typical symptoms of long COVID are fatigue, cough, and musculoskeletal pain, with muscle pain being one of the independent predictors of non-improvement in patients with long COVID, in addition to other symptoms of late covid in individuals who do not need hospitalization, but which can be debilitating for patients and significantly strain medical resources. As our sample had the majority of patients with the mild form of infection, only 3.7% required hospitalization, with the lowest prevalence of use of emergency services varying from 2.6% in Denmark, 6 months after diagnosis of SARS-CoV-2, numerous visits were recorded by individuals in consultations with a general practitioner, outpatient hospital consultations, emergency, and even hospital admissions, however, only a fraction of these individuals were treated in the hospital emergency [24].

However, there are studies that predict that their subjects, even with long COVID diagnosis, will recover without the help of specialists. In Brazil, there are many municipalities that do not have specialized services able to care for patients with late covid, making it necessary to restructure the health care network [25]. In the data collection by the Centers for Disease Control and Prevention (CDC) in partnership with Kaiser Permanente, one-third of patients (38%) sought specialized evaluation focusing mainly on behavioral/mental health (11%), and only (3%) of patients went to the pulmonologist [26].

In our study, after the adjusted analysis, individuals who described pain during and after SARS-Cov-2 infection were up to twice as likely to use the emergency care unit, in addition to doctors and specialized services, with neurologists being the most sought after of pulmonologists. Our sample was predominantly composed of women (59.6%), and in the use of health care, women sought more specialized services when compared to men, however, there was no increase in specialized medical care for patients after long COVID [27].

The results obtained in the present study must be interpreted, observing its limitations and potential. First, it is important to highlight that data collection covered the period from June to November 2021, making up six months after infection. The study had a representative sample, in which the majority (96.33%) of the participants did not require hospitalization and the diagnosis of covid-19 was made using the standard RT-PCR test performed between December 2020 and March 2021.

It is worth mentioning that this period does not coincide with the peak of cases of COVID-19 in Brazil and, therefore, must be considered when interpreting the data. Second, the covid-19 pandemic resulted in the prohibition of face-to-face collection by the ethics councils at the time of the study, therefore, individuals who were not located after five attempts at telephone contact, contact via the WhatsApp application and home visit were considered losses. Thirdly, lack of knowledge about the reasons for consultations and also about the study design stands out, which does not allow inferring causality, however, this problem was circumvented by including temporality in the questions. It is also noteworthy that the individuals were asked about the presence of symptoms, which reduces possible reporting biases. In addition, this study brings unprecedented data on the use of health services and musculoskeletal pain in individuals with long COVID in southern Brazil.

## Conclusions

All regions were related to the use of emergency care unit, specialist doctors, specialized services, cardiologist and neurologist. Except for the cervical region, all regions were related to the use of PHC. Regions related to emergency room use were thoracic region, upper limbs and body region. The cervical, upper limb and thoracic regions were related to the use of pulmonologist. Long COVID has affected part of the world's population and there is a huge lack of specific health care about this condition. It is necessary that social policies and health services are urgently reorganized for this new demand. Therefore, it is important to have knowledge of musculoskeletal pain during and after SARS-CoV-2 infection, because in this way we can quantify and offer subsidies for health professionals.

### Supplementary Information

**Additional file 1.**

## Data Availability

The datasets generated and/or analysed during the current study are not publicly available due the data are not public but are available from the corresponding author on reasonable request.
